# Diversity and distribution of extracellular microcrystals in holothuroid echinoderms

**DOI:** 10.1098/rsos.250514

**Published:** 2025-09-17

**Authors:** Kevin C. K. Ma, Fei Gao, Jean-François Hamel, Annie Mercier

**Affiliations:** ^1^ Department of Ocean Sciences, Memorial University, St. John's, Newfoundland and Labrador, Canada; ^2^ School of Marine Biology and Fisheries, Hainan University, Haikou, Hainan, People’s Republic of China; ^3^ Society for the Exploration and Valuing of the Environment (SEVE), St. Philips, Newfoundland and Labrador, Canada

**Keywords:** Apodida, biomineral, calcite, Dendrochirotida, uric acid, weddellite, sea cucumbers

## Abstract

Biomineralization research in echinoderms has been focused on skeletal structures, which provide strength and protection. Other minerals, specifically extracellular microcrystals, are described here for the first time in echinoderms. Six morphotypes were isolated from a holothuroid and classified into three chemical compounds. Uric acid crystals were associated with fluids from the hydrovascular system, perivisceral coelom and respiratory tree, while calcium carbonate crystals were detected in the epithelium of the respiratory tree and calcium oxalate dihydrate crystals were found in tissues of the cloaca, integument, Polian vesicle and tentacle. Uric acid crystals were mostly non-encapsulated in tissues, whereas, in fluids, they were often encapsulated by phagocytes, alone or in groups, suggesting that they are waste products. Calcium carbonate crystals in the respiratory tree and calcium oxalate dihydrate crystals in the cloaca suggest that they are being expelled from the body. Insofar as calcium oxalate dihydrate crystals could be involved in calcium regulation, we speculate that they might be crystallized and retained in ossicle-associated integumentary tissues and tentacles, which are susceptible to damage and could use them as a calcium reserve to synthesize and repair ossicles. Observations of microcrystals in different holothuroid species examined suggest their ubiquity in the class Holothuroidea and more generally, deuterostomes.

## 1. Introduction

Organisms can form solid substances through processes collectively known as biomineralization [1–3]. Biomineralization of organic precipitates and inorganic minerals (including more than 70 types) has been documented across many taxa, including microbes, fungi, plants and animals [2,4–7]. The ubiquitous production of biominerals is thought to be linked to the origin and maintenance of life on Earth [3,7,8]. The structure of these biominerals can be (mono)crystalline (i.e. hierarchically structured crystals), polycrystalline (comprising multiple crystalline parts oriented in different directions), mesocrystalline (a structure consisting of nanoparticles with well-defined shapes at micron- or macro-scales) or amorphous (i.e. non-crystalline solid) [9,10].

Biominerals of diverse morphologies and sizes may provide (i) mechanical and physical strength for cutting, grinding, forming skeletal structures and protecting against injuries and predators, (ii) components of sensory receptors (e.g. light, gravity and magnetic field) and (iii) reserves of calcium or other elements and ions [2,6,11,12]. In addition, biominerals can be by-products of biological activities such as metabolism and be pathological in diseased organisms [2,13–16].

The formation of biominerals can be biologically induced, biologically influenced or biologically controlled [4,5]. Biologically induced mineralization occurs as a product of biological activity (e.g. metabolism) and the indirect modification of the environment (e.g. chemistry favourable to mineral formation) [4,5]. In biologically influenced mineralization, organic matter (e.g. polysaccharides and proteins) can passively modulate crystal precipitation, shape and growth [5]. Finally, biologically controlled mineralization involves the uptake and the active incorporation of elements into structures through nucleation, growth and positioning of a mineral in an organism [4,5]. Controlled processes can be further classified relative to the site of mineral formation, chiefly into extra- and intra-cellular mineralization and more rarely inter-cellular mineralization [1,4,17].

In echinoderms, known minerals are mostly of biological origins, except for the bioaccumulation of zircon and ilmenite found in the digestive tract (diverticula) of some species [18]. Ossicles, plates, spines and other protective skeletal elements composed of calcium carbonate (calcite) are the most ubiquitous biominerals in echinoderms [2,6,11] and are formed through biological control [19]. A closer examination of hard parts made of calcium carbonate in echinoderms indicates that they (i) exhibit a nanocomposite/nanogranular architecture with individual mineral grains between 20 and 100 nm in diameter [19], (ii) are primarily constructed out of Mg-calcite (magnesium-rich, high-magnesium or magnesian calcite) [1] and (iii) develop as early as the larval stage [20–23]. Furthermore, teeth of echinoids (sea urchins) are composed of a combination of Mg-calcite and protodolomite—a mineral between calcite and dolomite [24,25]. When isolated, skeletal elements of Mg-calcite may be viewed with the naked eye (e.g. test plates in echinoids, dermal plates in some dendrochirotid holothuroids) [26] or be microscopic (e.g. dermal and podial ossicles).

Other types of biominerals found in echinoderms are crystalloids and amorphous minerals of unknown function and origin. Crystalloids are intra-cellular crystalline structures containing iron and sulphur that typically form thin arrays at or around sub-micron-scale [27–30]. Amorphous minerals are known to occur in four forms in echinoderms: hydrated ferric phosphate (iron- and phosphate-rich bodies), ilmenite (titanium-iron oxide), opaline silica (biogenic opal) and amorphous calcium carbonate [25,31–35].

Except for microscopic ossicles made of Mg-calcite [9,36,37], microcrystals (i.e. crystals ranging between 1 and 200 μm in size) [38] have not been explicitly reported and described in echinoderms. Notably, in the only series of publications related to their occurrence, the mere presence of calcium oxalate dihydrate (weddellite) and calcium fluoride (fluorite) in echinoderms was inconsistently listed, sometimes as unpublished data and never discussed [1,2,25,39]. With no further information, the existence of these crystals in echinoderms therefore remains ambiguous and unsubstantiated. Extracellular microcrystals of diverse morphologies (e.g. cuboids, rhombs, octahedrons) are reported from many other animal taxa, including calcium oxalate dihydrate crystals in sponges and tunicates [40,41], uric acid in arthropods [42] and a variety of crystal types in mammals [43–49]. Morphology (e.g. shape and colour) and solubility tests can be used to identify microscopic crystals found in urine of healthy and pathological individuals of mammals [14,16,48,50]. Acidic urine may contain crystals of uric acid, calcium oxalate, hippuric acid, sodium urate, calcium sulfate, cystine, leucine, tyrosine and cholesterol, whereas alkaline urine may contain triple phosphate, calcium carbonate, calcium phosphate and ammonium biurate [14].

During a study of the commercial sea cucumber *Cucumaria frondosa* (Holothuroidea: Echinodermata) in the North Atlantic, several types of extracellular microcrystals standing out from the typical ossicles were detected. They were systematically surveyed across different tissues and fluids to examine their morphology and preliminary composition and to provide the first definitive catalogue of extracellular microcrystals in an echinoderm. The possible roles and transport pathways of some microcrystals were also explored and their occurrence in other species of sea cucumber was assessed.

## 2. Material and methods

### 2.1. Collection

Between 2021 and 2024, adult individuals of the holothuroid echinoderm *C. frondosa* were collected through SCUBA at depths between 7 and 12 m from Mobile Bay (47°14'45"N; 52°50'13"W), Torbay (47°39'54"N; 52°43'36"W) and Tors Cove (47°12'44"N; 52°50'39"W), off the coast of insular Newfoundland, eastern Canada. Live individuals were held in flow-through seawater tanks (flow rates: 300−450 l min^–1^; tank volumes: 400−650 l) in the laboratory at the Department of Ocean Sciences of Memorial University (47°37'31"N; 52°39'46"W) until they were examined. The whole wet weight of these individuals ranged from 36 to 344 g (mean of 180 g; *n* = 100).

### 2.2. Sample processing

In the search for microcrystals, samples (*n* = 3−6 per tissue type) were taken from the following locations: ampulla, cloaca, genital papilla (female and male), gonad (female and male), gonoduct (female and male), integument of the body wall (i.e. tissue without muscle bands; bivium and trivium), integument of the introvert (i.e. the collar of tissue between the tentacles and body wall), intestine, madreporite, podia (bivium and trivium), Polian vesicle (i.e. the membrane), radial and ring canals (both canals combined as one sample), respiratory tree, muscles (circular, longitudinal and retractor), stomach, stone canal, tentacle and vesicle of the tentacle. In addition, coelomocyte aggregates extracted from the perivisceral coelomic fluid and coelomocyte aggregates extracted from the Polian vesicle, which were considered as tissues, were also analysed (*n* = 4 samples per tissue/aggregate type). These tissue samples (including aggregates) were rinsed with distilled water for approximately 10 s to minimize cross-contamination from surrounding fluids. Subsequently, samples were digested in a solution of sodium hypochlorite (8.25%). The next day, they were examined under a light microscope (Nikon Eclipse 80i, Japan).

Because preliminary examinations revealed that sodium hypochlorite (an alkaline solution) could chemically interact with some crystal types, another series of fresh (i.e. undigested) samples (*n* = 3−21 per tissue type) were collected from new individuals. Fresh samples were analysed from all tissues (including aggregates) described above and from the following fluid samples: fluid expelled out of the body from the respiratory tree, fluid from the Polian vesicle, fluid from the radial and ring canals, fluid from the respiratory tree, fluid from the vesicle of the tentacle, mucus (discharged externally) and perivisceral coelomic fluid. The tissue samples were rinsed with distilled water for approximately 10 s, cut into small thin pieces with a scalpel where applicable and immediately (within 10 min) examined for microcrystals under the light microscope (see above). In addition to the tissue and fluid samples, fresh materials from the digestive tract (i.e. intestinal content) were analysed (*n* = 3 samples of intestinal content from different individuals). Prevalence of microcrystals (presence or absence) in each of the examined tissues and fluids was determined. A prevalence of ≤33% of samples examined was considered low, >33–66% was considered moderate and >66% was considered high.

### 2.3. Identification of crystals

Crystals detected under the light microscope were photographed with a digital camera (Olympus DP73, Japan). The shape of crystals was used to first characterize them (e.g. cuboidal, barrel-shaped, rhombic, rhombohedral, rosette-shaped, cylindrical, octahedral, dodecahedral). The terminology used to name the various microcrystal morphologies was modified from Neuendorf [16], Ord [51], Baconnier *et al.* [52] and Daudon *et al.* [53,54]. Their size (i.e. including the length of the longest edge for cuboidal crystals or the distance between two distal ends for all other types of crystals) was determined from images using ImageJ (version 1.53 k). For rhomb-shaped crystals, one of the interior angles (see Prien and Frondel [55]) was determined using ImageJ for a subset of crystals.

Complementary morphological analyses were made by imaging target microcrystals with a scanning electron microscope (SEM; PHENOM Pro and FEI MLA 650F). From samples with confirmed presence of microcrystals under a light microscope, cuboidal, rhombic, cylindrical, octahedral and dodecahedral crystals that were suspended in solution (either in sodium hypochlorite for digested samples or in bodily fluids for undigested samples) were transferred onto an SEM sample stub. The wet samples were dried for a period of approximately 1 day in a semi-enclosed container surrounded by a desiccant (silica gel) at ambient temperature (*n* = 12 prepared samples of a mix of cuboidal and rhombic crystals; *n* = 12 samples of cylindrical crystals; *n* = 12 samples of a mix of octahedral and dodecahedral crystals). Moreover, elemental compositions were determined by emitting X-ray energies on the surface of crystals using the FEI MLA 650F SEM energy-dispersive X-ray (EDX) spectroscopy detectors (Bruker) [56–59].

Chemical compositions of targeted microcrystals were assessed using Raman spectroscopy (Renishaw inVia confocal Raman microscope). Similar to how crystal samples were prepared for SEM, crystals suspended in solution were transferred onto a microscope slide (without a cover slip) and dried (see above). Triplicates of each type of crystal—cuboidal, rhombic, cylindrical, octahedral and dodecahedral crystals—were analysed.

Less morphologically complex crystals (e.g. cuboidal and rhombic forms can be difficult to identify based only on morphology) were subjected to complementary solubility tests to support their chemical identity based on basic (alkaline) or acidic functional groups and heated (in solution) to assess their thermal properties, as per Lee *et al.* [48]. Between 15 and 20 microcrystals (a mix of cuboidal and rhombic crystals) were used in each test. For the first test, crystals were exposed to a solution of sodium hypochlorite (8.25%) and examined after 5, 15 and 20 min. For the second test, crystals were exposed to ethanol (75%), which was slightly acidic and examined after 24 h. For the third test, crystals immersed in hydrovascular fluid were exposed to heat (50–55°C) in a drying oven and examined after 1, 16 and 24 h. The absence or eventual disappearance of these crystals indicated solubility (i.e. dissolution of crystals in a liquid solvent) and their continued presence indicated insolubility.

To better interpret the solubility tests and to characterize the baseline pH level within the body of *C. frondosa*, the pH of the following was determined using a benchtop pH meter (Accumet AB150, Fisher Scientific): seawater from the ocean, distilled water, 8.25% sodium hypochlorite, 75% ethanol, fluids from the perivisceral coelom, Polian vesicle, radial and ring canals, respiratory tree and vesicle of the tentacle, mucus (discharged externally) and fluid expelled out the body from the respiratory tree. Triplicates (volume: 5 ml) of each type of fluid (each from different individuals) were tested.

### 2.4. Encapsulation of crystals

Fresh tissue and fluid samples with confirmed presence of microcrystals (see above on undigested tissue and fluid samples) were examined for the presence or absence of a thin envelope surrounding these crystals under the light microscope (see above). The number of encapsulated and non-encapsulated crystals was determined.

### 2.5. Examination of non-biological sources of crystals

To detect potential non-biological sources of crystals created by chemical reactions that may have contaminated samples during processing, we transferred approximately 50 ml of seawater, distilled water and 8.25% sodium hypochlorite into a glass beaker (*n* = 3 samples per fluid type). All three fluids were examined for crystals under a light microscope (as described above) within 10 min.

As the presence of ossicles in the tissues examined could facilitate the non-biogenic formation of calcium-based microcrystals (i.e. not occurring naturally in fluids and tissues) [60,61], additional controls were prepared, consisting of a 50 ml mixture in a glass beaker of: (1) seawater and a higher concentration of sodium hypochlorite (1:1 ratio), (2) seawater and a lower concentration of sodium hypochlorite (3:1 ratio) and (3) seawater and sodium hypochlorite (1:1 ratio) with about 500 mg of ossicles—a source of Mg-calcite extracted from sea cucumbers. The presence of crystals from these solutions was examined within 10 min and after 5, 12, 19, 26 and 40 days.

### 2.6. Supplementary observations

To confirm that the occurrence of extracellular microcrystals was not restricted to a specific sea cucumber population or to only one species, additional assessments of the presence of microcrystals were carried out on (i) individuals of *C. frondosa* from shallow waters (approx. 2 m depth) around Qikiqtait, Nunavut, northern Canada (56°37'48"N; 79°12'30"W), (ii) individuals of *C. frondosa* and another dendrochirotid holothuroid species, *Psolus phantapus*, from the two fishing areas of St. Pierre Bank off the south coast of Newfoundland (western area: south of 46°30'N and west of 56°28'W; eastern area: south of 46°30'N and east of 56°08'W), (iii) individuals of the dendrochirotid holothuroid *Psolus fabricii* from Logy Bay (47°37'31"N; 52°39'48"W) and Tappers Cove (47°39'54"N; 52°43'37"W), Newfoundland and Labrador, Canada and (iv) individuals of the apodid holothuroid *Chiridota laevis* from waters off the coast of Pembroke, Maine, eastern United States of America (44°53'50"N; 67°06'40"W). Selected body components from these supplemental individuals were examined for extracellular microcrystals (see §2.2 on how undigested and digested tissues were processed): cloaca, integument of the body wall, perivisceral coelomic fluid, Polian vesicle and its fluid, respiratory tree, retractor muscle and tentacle.

## 3. Results

### 3.1. Morphological diversity of microcrystals

#### 3.1.1. Extracellular microcrystals

**Figure 1.**
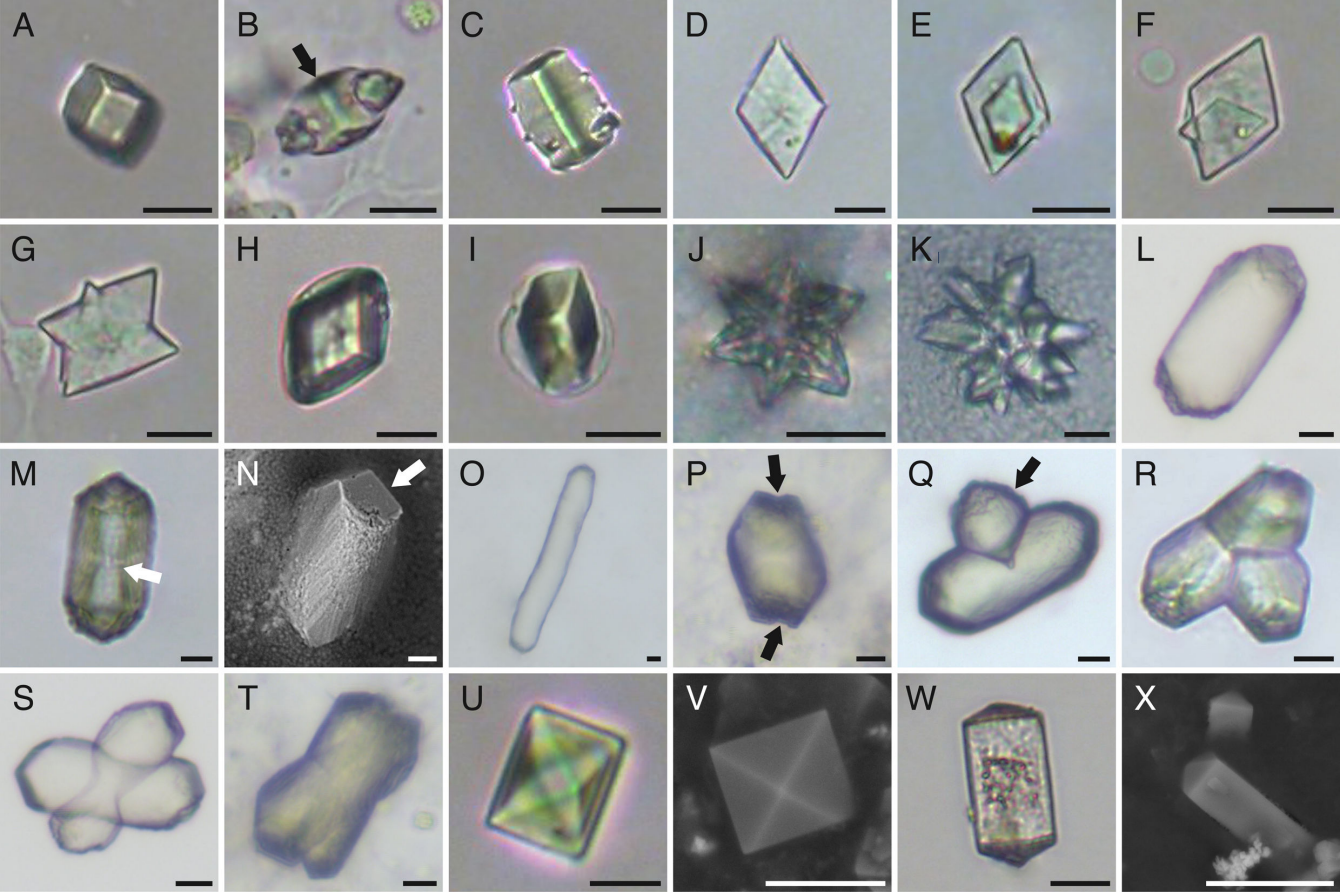
Microcrystals in *C. frondosa*: (A–B) cuboidal crystal (arrow indicates the line similar to barrel-shaped crystals), (C) barrel-shaped crystal with line along the length of the structure, (D) rhombic crystal, (E) multi-layered rhombic crystal, (F–G) other variants of the rhombic crystal, (H–I) rhombohedral crystal, (J) small rosette-shaped crystal, (H) large rosette-shaped crystal, (I) cylindrical crystal, (M) cylindrical crystal with visible internal structure (arrow), (N) cylindrical crystal with faceted ends (arrow), (O) unusually elongated cylindrical crystal, (P) cylindrical crystal with notches (arrows), (Q) cylindrical crystal with a lateral outgrowth (arrow), (R) three-pointed crystal, (S–T) cross-shaped crystal, (U–V) octahedral crystal and (W–X) dodecahedral crystal. Microcrystals in panels N, V and X imaged using a scanning electron microscope and all other microcrystals imaged under a light microscope. Scale bars represent 10 µm.

**Figure 2.**
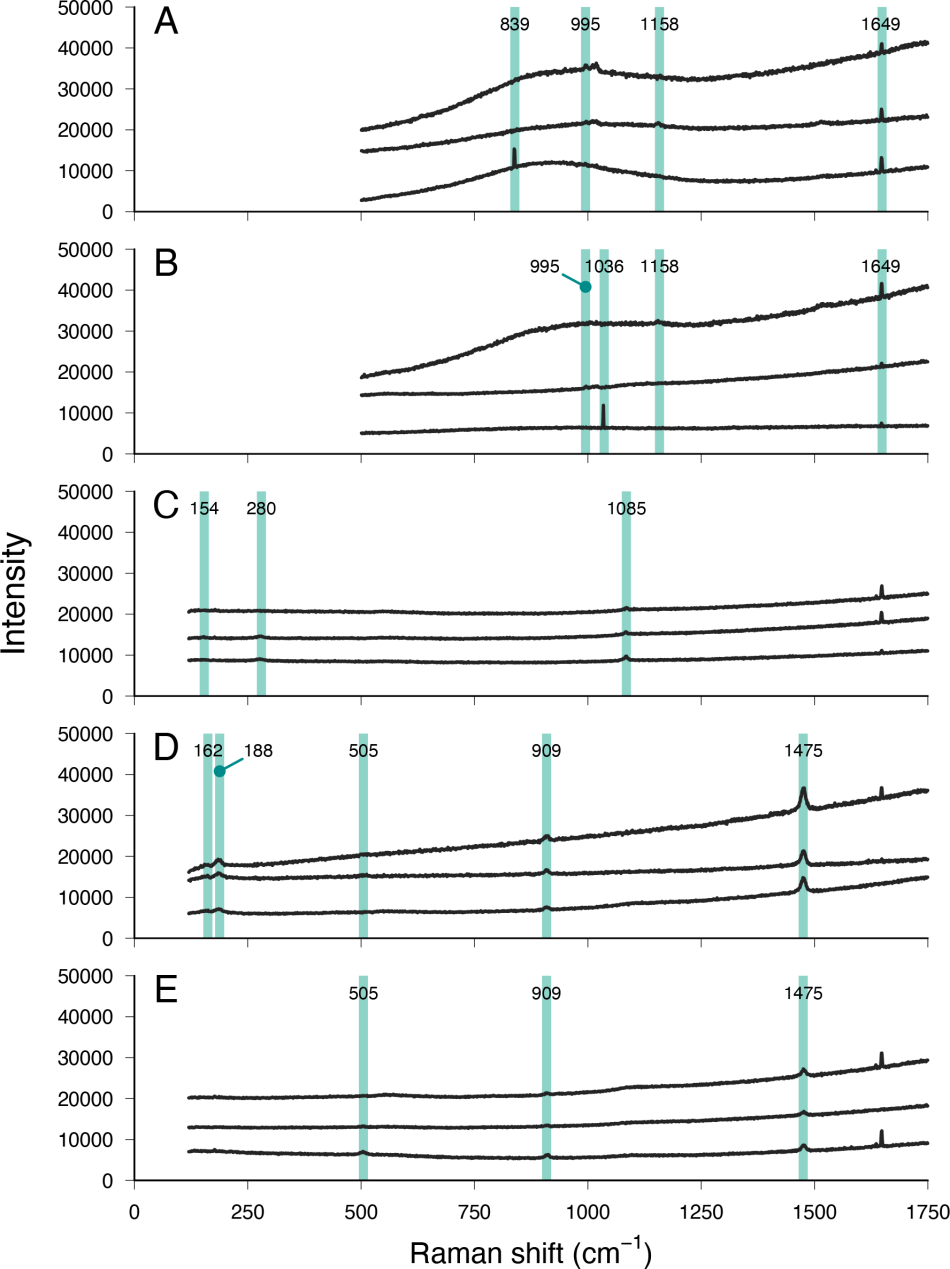
Raman spectroscopy spectra of five morphologies of microcrystals isolated from *C. frondosa*. Morphologies: (A) cuboidal, (B) rhombic, (C) cylindrical, (D) octahedral and (E) dodecahedral. Green bands highlight diagnostic peaks characteristic of uric acid in A and B, of calcium carbonate in C, and of calcium oxalate dihydrate in D and E.

Multiple morphologies of extracellular microcrystals (cuboidal, barrel-shaped, rhombic, rhombohedral, rosette-shaped, cylindrical, cross-shaped and related variants, octahedral and dodecahedral) were identified from tissue and fluid samples of individuals of *C. frondosa* (figure 1) and grouped into six morphotypes (table 1). A group of commonly co-occurring microcrystals, consisting of simple morphologies (cuboidal, barrel-shaped, rhombic and rhombohedral) to more complex ones (rosette-shaped) were detected (figure 1A–K). Cuboidal (figure 1A–B) and barrel-shaped crystals (figure 1C) were colourless, three-dimensional, 3−20 µm in length (longest edge; table 1). Some cuboidal and most barrel-shaped crystals exhibited a line along the length of the crystalline structure (figure 1B–C). Rhombic crystals and related variants (figure 1D–G) were translucent, thin and flat, between 5 and 29 µm in length (table 1), sometimes rhombohedral in shape (i.e. thicker form of the rhombic crystal of similar size; figure 1H–I), characterized in the present case by one of the interior angles being 62 ± 6° on average (±s.d.; *n* = 97). Rosette-shaped crystals (figure 1J–K) were colourless, star-shaped with six or more points and between 19 and 47 µm across (table 1). Cuboidal and rhombic shapes were soluble in sodium hypochlorite (within 5 min; pH of approx. 12.90) or when heated (within 1 h; 50−55°C), and insoluble in ethanol (within 24 h; pH of approx. 6.44) or in internal bodily fluids with pH ranging between 7.55 and 7.73 (electronic supplementary material, table S1). Although no elemental data are available because these crystals were resistant to SEM procedures, cuboidal and rhombic crystals were successfully analysed using Raman spectroscopy. The Raman spectra of these crystals exhibited peaks at 839, 995, 1036, 1158 and/or 1649 cm^–1^ (figure 2A–B).

**Table 1.** Size of microcrystals detected in *C. frondosa* comprising multiple morphologies grouped into six corresponding morphotypes. Minimum and maximum sizes provided along with mean, standard deviation (s.d.), and sample size (*n*).

morphotypes (chemical compound)	min-max size (µm)	mean size ± s.d. (µm)	*n*
cuboidal–barrel-shaped (uric acid)	2.5–20.1	7.5 ± 2.8	143
rhomb-shaped (uric acid)	4.5–28.9	14.7 ± 4.3	352
rosette-shaped (uric acid)	19.4–47.3	36.7 ± 11.5	5
cylindrical and related variants (calcium carbonate)	11.8–141.9	38.1 ± 19.8	178
octahedral (calcium oxalate dihydrate)	4.8–89.0	24.8 ± 10.8	457
dodecahedral (calcium oxalate dihydrate)	7.9–49.7	24.5 ± 11.4	90

**Figure 3.**
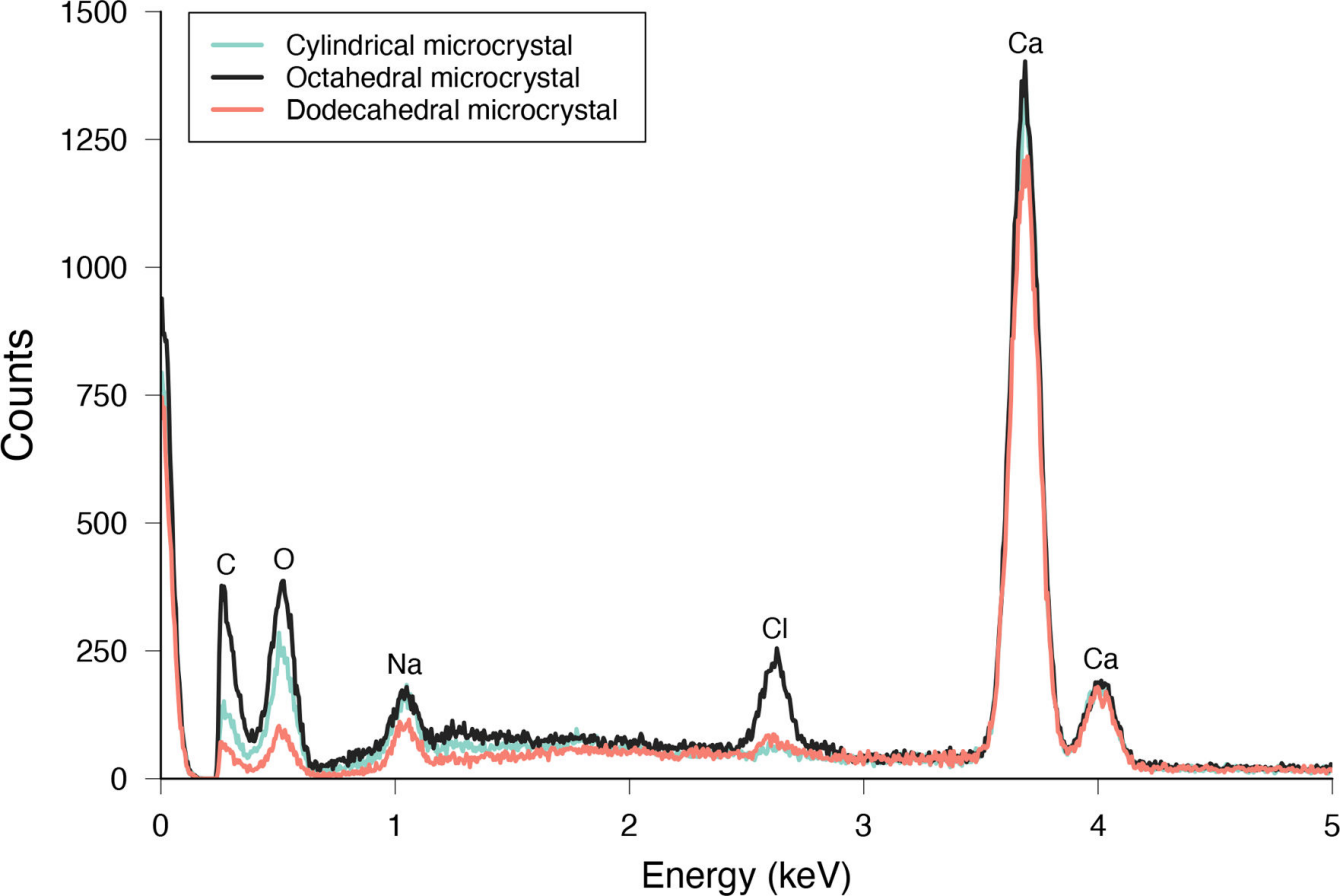
Energy-dispersive X-ray spectroscopy spectra of three morphologies of microcrystals isolated from *C*. *frondosa*. Morphologies: cylindrical (green line), octahedral (black line) and dodecahedral (red line). C = carbon, O = oxygen, Na = sodium, Cl = chloride, Ca = calcium.

Another group of similar microcrystals, consisting chiefly of a cylindrical morphology of varying lengths (figure 1L–O) and some other derivative forms (e.g. notched ends, three-pointed-shaped and cross-shaped; figure 1P–T), were observed. These crystals were three-dimensional, translucent and between 12 and 142 µm in length (table 1). The internal structure of this microcrystal was dumbbell-shaped (figure 1M). The ends were faceted, which was clearly visible in the SEM image (figure 1N). EDX analysis revealed that these microcrystals were composed of calcium with carbon and oxygen (figure 3). Unlike ossicles, which contain high levels of magnesium, EDX analysis did not detect any spectral signal associated with magnesium (i.e. 1.253 keV). Likely from a source of salt contamination, sodium and chloride were often detected in EDX analysis of these crystals (figure 3). Raman spectra of cylindrical crystals showed peaks at 154, 280, 1085 and/or 1649 cm^–1^ (figure 2C).

A third group of related microcrystals was observed, consisting of two distinct morphologies, octahedral and dodecahedral (figure 1U–X), the former being more common than the latter. These two morphologies tended to co-occur and have pyramidal structural components, which were evident through SEM imaging (figure 1V,X). Octahedral crystals were three-dimensional, transparent, bi-pyramidal in shape and between 5 and 89 µm across (table 1 and figure 1U–V). Dodecahedral crystals were three-dimensional, transparent, rectangular prisms with the bases of two square pyramids on opposite ends of the prism and between 8 and 50 µm in length (table 1 and figure 1W–X). EDX analysis of octahedral and dodecahedral microcrystals showed that they consisted of calcium with carbon and oxygen (figure 3). As with the analysis on cylindrical crystals, sodium and chloride were often detected in EDX analysis of octahedral and dodecahedral crystals (figure 3). Raman spectra of octahedral and dodecahedral crystals presented with peaks at 182, 188, 505, 909, 1475 and/or 1649 cm^–1^ (figure 2D–E).

#### 3.1.2. Non-biogenic microcrystals

Two types of crystals were observed in control solutions: thin quadratic or rectangular crystals and peanut-shaped crystals. Quadratic or rectangular crystals (sodium chloride) [62] were commonly encountered in both control solutions and biological samples. Peanut-shaped crystals (monohydrocalcite) [63] were found in some of the control solutions after 5 days and in all three types of fluid mixtures of seawater and sodium hypochlorite (including those with ossicles) after 12 days. Therefore, observations of these two types of crystals from fluid and tissue samples of *C. frondosa* were excluded from this study.

### 3.2. Corporeal distribution of microcrystals

**Figure 4.**
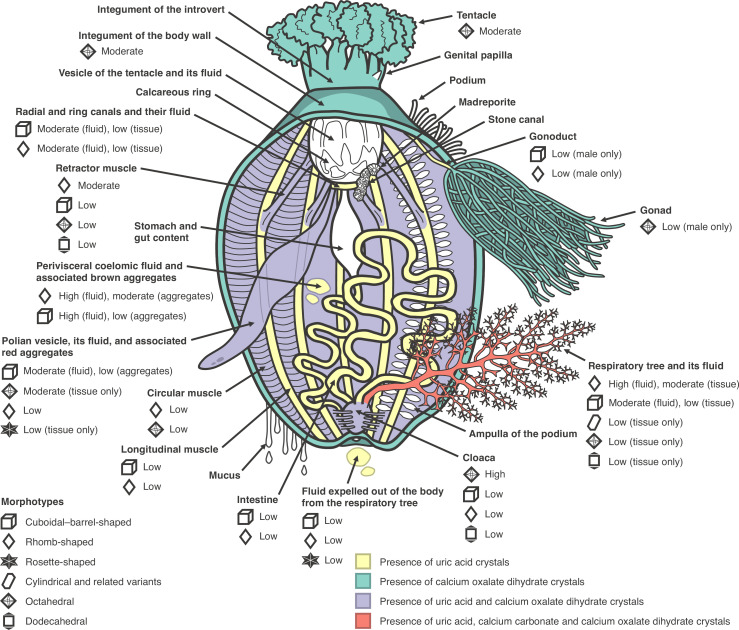
Corporeal distribution of microcrystals in *C. frondosa* comprising multiple morphologies grouped into six morphotypes and three chemical compounds (uric acid, calcium carbonate and calcium oxalate dihydrate). Low = prevalence of ≤33% of the samples examined per body component, moderate = prevalence of ˃33−66% of the samples, high = prevalence of ˃66% of the samples.

The occurrence and prevalence of six morphotypes of crystals varied among different tissues and fluids within the body of *C. frondosa* (figure 4 and electronic supplementary material, table S2). Two morphotypes—(i) the cuboidal and barrel-shaped (i.e. an elongated morphology of the cuboidal form) and (ii) rhomb-shaped (e.g. rhombic, rhombohedral)—were present (a) in the fluid expelled out the body from the respiratory tree, fluid from the perivisceral coelom, fluid from the Polian vesicle, fluid from the radial and ring canals and fluid from the respiratory tree, (b) among coelomocyte aggregates from the perivisceral coelom and coelomocyte aggregates from the Polian vesicle, and (c) in tissues of the cloaca, intestine, male gonoduct, radial and ring canals, respiratory tree and retractor muscle (figure 4). In addition, the rhomb-shaped morphotype was detected in the tissue samples of the circular and longitudinal muscles and the Polian vesicle (figure 4).

Among the body tissues and organs in which they were observed, the prevalence of the cuboidal–barrel-shaped and the rhomb-shaped morphotypes was moderate to high in the fluid from the perivisceral coelom (i.e. ˃33% of samples examined), Polian vesicle, radial and ring canals and respiratory tree and in the tissue from the respiratory tree and retractor muscle (figure 4 and electronic supplementary material, table S3). Cuboidal–barrel-shaped, rhomb-shaped and rosette-shaped morphotypes were observed in the fluid expelled out of the body from the respiratory tree and in the tissue of the Polian vesicle (figure 4). The prevalence of rosette-shaped morphotype in both was low (i.e. ≤33% of samples examined; figure 4). The cylindrical morphotype (including cross-shaped and other related variants) was present in the tissue of the respiratory tree, where its prevalence was low (figure 4 and electronic supplementary material, table S3). The octahedral and dodecahedral morphotypes were present in the tissues of the cloaca, respiratory tree and retractor muscle (figure 4 and electronic supplementary material, table S3). The octahedral morphotype was also detected in tissues of the tentacle, circular muscle, integument of the body wall (bivium and trivium), male gonad and Polian vesicle (figure 4). The prevalence of this morphotype was relatively high (>66% of samples examined) in the cloaca and moderate (>33–66%) in the integument of the body wall (bivium and trivium), Polian vesicle and tentacle compared with all other body components associated with this microcrystal morphotype. The prevalence of the dodecahedral morphotype was low (≤33%) in all associated tissues and organs (figure 4 and electronic supplementary material, table S3).

No microcrystals were found in the following tissues: ampulla of the podium, calcareous ring, female gonad, female gonoduct, genital papilla (male and female), integument of the introvert, madreporite, podium, radial canal, ring canal, stomach, stone canal and vesicle of the tentacle. Moreover, no microcrystals were found in the mucus, intestinal content or fluid from the vesicle of the tentacle (electronic supplementary material, table S2).

### 3.3. Encapsulated microcrystals

**Figure 5.**
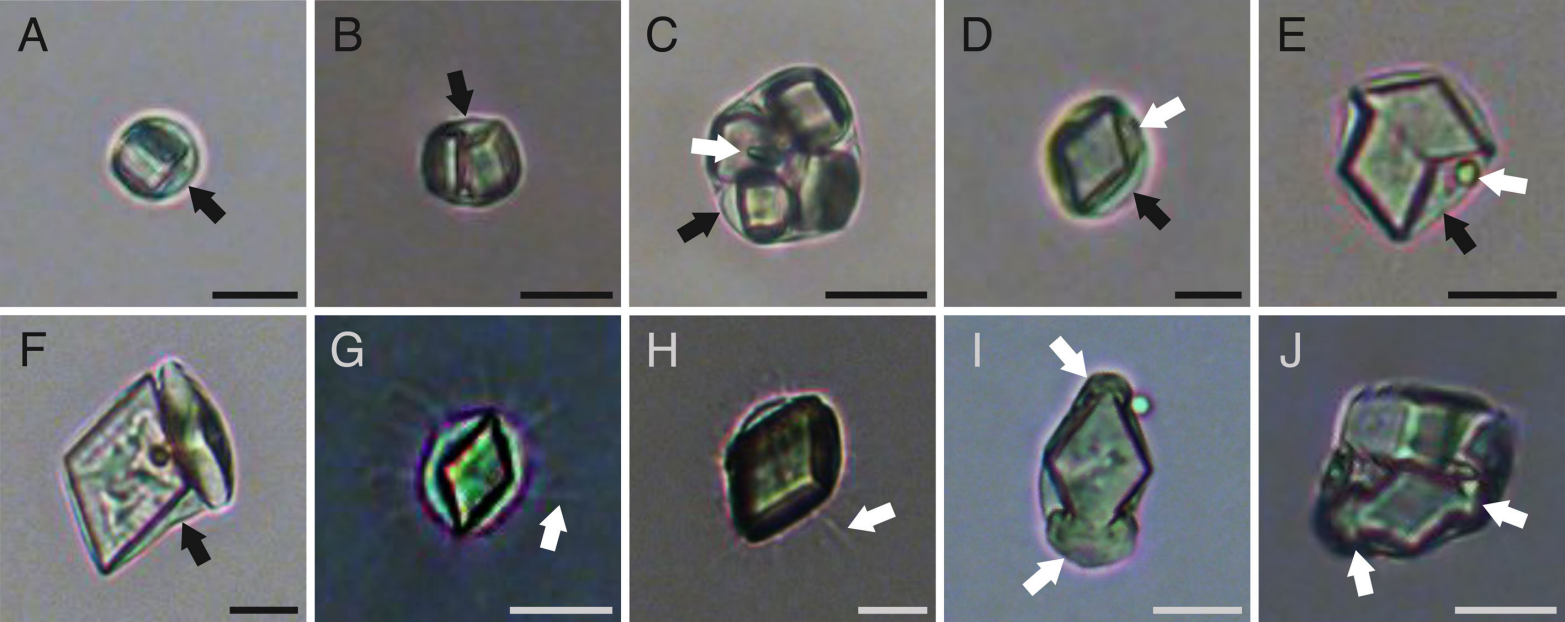
Microcrystals encapsulated by phagocytic coelomocytes of *C. frondosa*: (A) one cuboidal microcrystal, (B) two cuboidal microcrystals, (C) multiple cuboidal microcrystals, (D) one rhomb-shaped microcrystal, (E) two rhomb-shaped microcrystals, (F) orthographic view of a rhomb-shaped microcrystal and oblique view of a another, (G–H) rhomb-shaped microcrystal inside a phagocytic coelomocyte with filopodia (arrow), (I) rhomb-shaped microcrystal packaged with other materials around the distal ends of the crystal (arrows) and (J) several microcrystals packaged with other material (arrows). Black arrows in panels A–F indicate the phagocyte, white arrows in panels C–E indicate a foreign particle. Scale bars represent 10 µm.

Based on fresh undigested samples, non-encapsulated cuboidal and rhomb-shaped (i.e. rhombic and rhombohedral) microcrystals were typically found in tissues (prevalence of 54−100% of crystals examined). When observed in bodily fluids these microcrystals were seen encapsulated in phagocytic coelomocytes (figure 5). In several instances, a single phagocyte was seen to engulf more than one microcrystal (figure 5B–C,E–F,J). In the perivisceral coelomic fluid, encapsulated crystals were sometimes observed inside phagocytes with visible filopodia (figures 1E and 5G–H). In addition, we found several examples of visible packaging of other materials (possibly foreign) adjacent to these crystals within the same phagocyte in the perivisceral coelomic fluid and in the fluid from the radial and ring canals (figure 5I–J).

Although cuboidal and rhomb-shaped crystals were present in a variety of body components, those that were encapsulated were only detected (i) in the fluid expelled out of the body from the respiratory tree and in the fluids from the perivisceral coelom, Polian vesicle, radial and ring canals and respiratory tree, (ii) among coelomocyte aggregates from the Polian vesicle, and (iii) in tissues of the intestine and respiratory tree (table 2). The prevalence of encapsulated cuboidal and/or rhomb-shaped microcrystals, when present, was low to moderate (0−46% of crystals examined) in tissues, moderate to high (33−100%) in fluids and high (67−75%) among coelomocyte aggregates (table 2).

**Table 2.** Occurrence (present or not detected) and prevalence (proportion of total microcrystals) of encapsulated microcrystals in the various tissues and fluids of *C. frondosa*. Numerator of the fraction = number of encapsulated microcrystals observed, denominator of the fraction = total number of microcrystals examined.

body component	occurrence of encapsulated crystals	prevalence of encapsulated cuboidal crystals (fraction)	prevalence of encapsulated rhomb-shaped crystals (fraction)
tissues			
circular muscle	not detected		
cloaca	not detected		
intestine	present	25% (1/4)	20% (1/5)
longitudinal muscle	not detected		
male gonoduct	not detected		
polian vesicle	not detected		
radial and ring canals	not detected		
respiratory tree	present	45% (5/11)	46% (17/37)
retractor muscle	not detected		
soft tissues			
coelomocyte aggregates from perivisceral coelom	not detected		
coelomocyte aggregates from Polian vesicle	present	67% (2/3)	75% (3/4)
fluids			
fluid expelled out the body from respiratory tree	present	33% (1/3)	64% (30/47)
fluid from perivisceral coelom	present	45% (15/33)	68% (51/75)
fluid from Polian vesicle	present	35% (6/17)	77% (20/26)
fluid from respiratory tree	present	83% (5/6)	100% (14/14)
fluid from radial and ring canals	present	43% (12/28)	34% (49/143)

### 3.4. Supplementary observations

As with the nearshore populations (Torbay and Tors Cove; see above), crystals were detected in individuals from offshore populations of *C. frondosa* from the St. Pierre Bank. Specifically, rhomb-shaped crystals were observed in the tissues of the cloaca, respiratory tree and retractor muscle and octahedral crystals in the tentacles and integument of the body wall (trivium). Rhomb-shaped crystals were found in the fluid from the Polian vesicles in *C. frondosa* collected from Nunavut. In the sea cucumber *Psolus fabricii*, rhombic crystals and related variants were present in the perivisceral coelomic fluid. Octahedral crystals were detected in the tentacles of *Psolus phantapus* and in the integument of the body wall (bivium and trivium not distinguished) of *Chiridota laevis*.

## 4. Discussion

**Figure 6.**
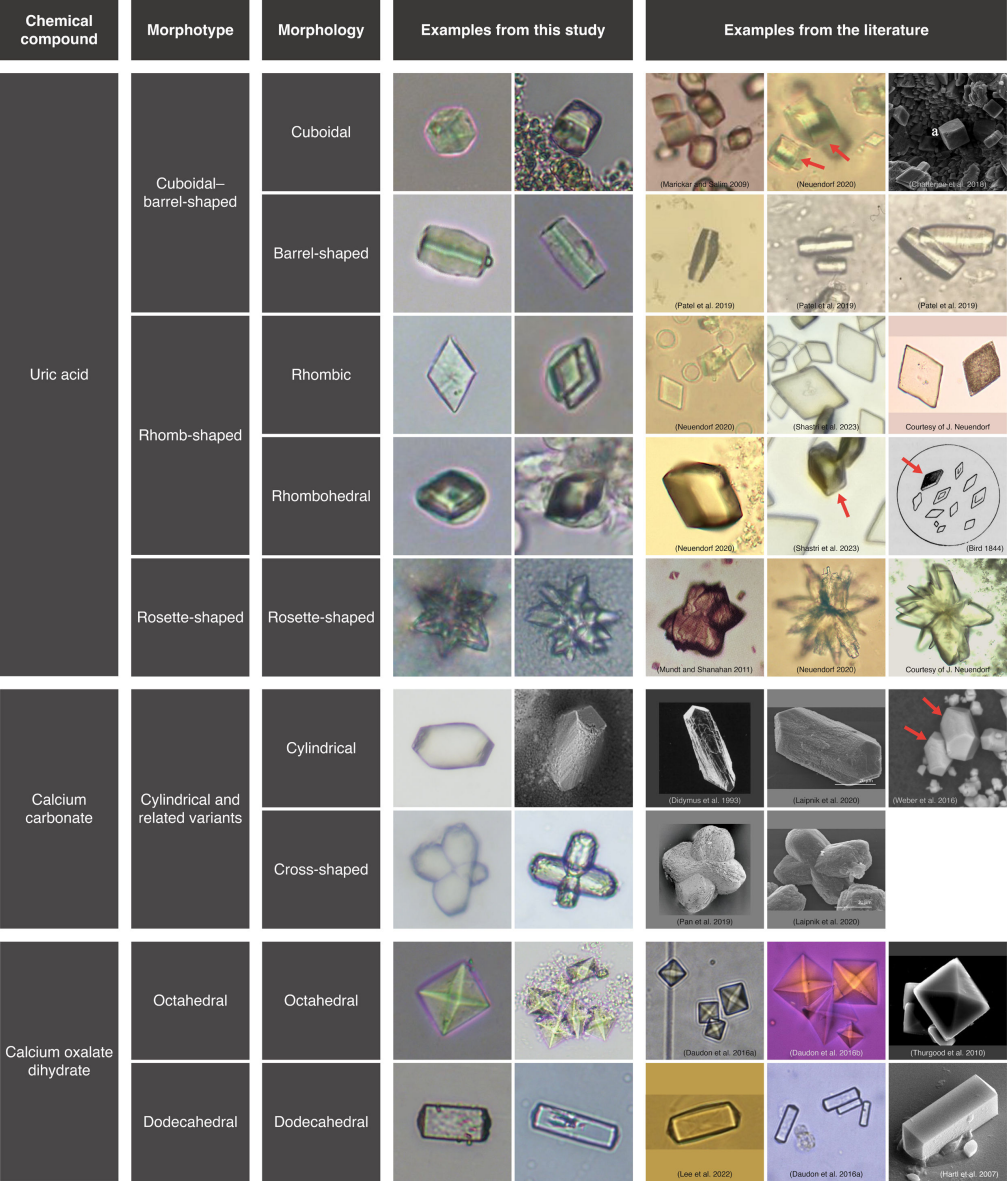
Morphologies of microcrystals in *C. frondosa* (this study) compared with non-echinoderm or laboratory-grown examples from the literature. Images from the literature were adapted from Mundt and Shanahan [14], Patel *et al.* [15], Neuendorf [16], Lee *et al*. [48], Daudon *et al*. [53,54], Bird [64], Free [67], Marickar and Salim [68], Chatterjee *et al*. [69], Shastri *et al*. [75], Didymus *et al.* [80], Weber *et al*. [85], Pan *et al.* [87], Laipnik *et al*. [88], Thurgood *et al*. [91], Hartl *et al*. [94]. NB: barrel-shaped crystals described by Marickar and Salim [68] were classified as cuboidal to better fit this morphological comparison.

Extracellular microcrystals exhibiting distinct morphologies were documented for the first time in the phylum Echinodermata. Diagnostics were made of the microcrystals found in the holothuroid *C. frondosa* based on those reported in other phyla (see summaries in table 3 and figure 6), suggesting that their synthesis is conserved at least among the superphylum Deuterostomia, which includes echinoderms and chordates. The commonly observed microcrystals in holothuroid tissues and fluids were the cuboidal and rhombic shapes. Strictly based on their morphology, they are analogous to crystals described in crustaceans [42] and mammals [16,67–70,75]. Barrel-shaped, rosette-shaped and rhombohedral microcrystals showed similar morphological features as those described in humans, including the characteristic line along the length of the barrel-shaped type [14–16,70]. Moreover, the complexity of rosette-shaped types may be a function of size in *C. frondosa* (smaller crystals being less complex, i.e. less slender and fewer protrusions, relatively flattened; figure 6). Cylindrical crystals with faceted ends were reported by Didymus *et al*. [80], Weber *et al*. [85] and Laipnik *et al*. [88]. Despite being morphologically different, the cross-shaped crystals (with their variants; figure 6) were suspected to be resulting from a natural morphing process called slip (sliding of a part of the crystal) or twinning (two or more intergrown crystals) [95,96].

**Table 3.** Varieties of microcrystals detected in *C. frondosa* and diagnostic characteristics of microcrystals observed in non-echinoderm taxa or grown under laboratory conditions.

morphotypes	chemical compound (molecular formula)	characteristics	references
cuboidal–barrel-shaped	uric acid (C_5_H_4_N_4_O_3_)	morphologies include cuboidal (cubes, cubic) and barrel-shaped crystals with characteristic line along the length of crystal structure; cuboidal crystals sometimes described as barrel-shaped or rhomboidal block; cuboidal crystals approximately 20 µm in size	Patel *et al*. [15], Neuendorf [16], Ord [51], Bird [64], Mitchell [65], Tyson [66], Free [67], Marickar and Salim [68], Chatterjee *et al*. [69]
rhomb-shaped	uric acid (C_5_H_4_N_4_O_3_)	morphologies include rhomb-shaped (rhombic, rhombohedral, diamonds or lozenges) and rhomboidal (whetstone-, football- or lemon-shaped) crystals; variants include multi-layered rhombic crystals and outgrowth at surface of rhomboidal crystals; rhomboidal crystals 130–190 µm in size; rhomb-shaped crystals 18–34 µm and one of interior angles 65°; soluble in alkali or when heated and insoluble in alcohol, acetic acid and hydrochloric acid; thin crystals generally colourless and thicker ones can be stained yellow, amber or brown by urine	Mundt and Shanahan [14], Neuendorf [16], Lee *et al*. [48], Fogazzi *et al*. [50], Daudon *et al*. [53], Prien and Frondel [55], Bird [64], Mitchell [65], Tyson [66], Marickar and Salim [68], Chatterjee *et al*. [69], Kleeberg *et al*. [70], Freitag and Hruska [71], Frochot and Daudon [72], Sienes Bailo *et al*. [73], Torous *et al*. [74], Shastri *et al*. [75]
rosette-shaped	uric acid (C_5_H_4_N_4_O_3_)	depiction of crystal in the literature variable; no size information	Mundt and Shanahan [14], Neuendorf [16], Lee *et al*. [48], Ord [51], Bird [64], Tyson [66], Kleeberg *et al*. [70]
cylindrical and related variants	calcium carbonate (CaCO_3_)	morphologies include cylindrical (laminar calcite, rod-shaped or pillar-shaped) and cross-shaped crystals and other variants (star- and cauliflower-shaped); cylindrical crystals with shorter body may be described as polyhedral; cylindrical crystals morphologically resemble two other calcium carbonate structures: pineal deposits (10–20 µm in size) and otoconia (5–45 µm); deformed otoconia can also be cross-shaped (approx. 9 µm); cylindrical crystals 10–71 µm in size, cross-shaped crystals 19–53 µm in size	Díaz-Espiñeira *et al.* [44,76], Baconnier *et al*. [52], Ross and Peacor [77], Johnsson *et al*. [78], Ross and Pote [79], Didymus *et al.* [80], Dominguez-Vera *et al*. [81], Jang *et al*. [82], Kniep [83], Silva-Castro *et al*. [84], Weber *et al*. [85], Zhang *et al*. [86], Pan *et al*. [87], Laipnik *et al*. [88], Nawarathna *et al*. [89]
octahedral	calcium oxalate dihydrate (C_2_H_4_CaO_6_⋅2H_2_O)	described as tetragonal bi-pyramidal (or di-pyramidal) or envelope-shaped crystals with two intersecting diagonal lines or planes; variants include cross-shaped and flower-like crystals; octahedral crystals 4–93 µm in size; soluble in hydrochloric acid, nitric acid, and sodium hydroxide and insoluble in acetic acid; colourless	Mundt and Shanahan [14], Neuendorf [16], Saffo and Lowenstam [40], Tawashi *et al*. [43], Keller and Goddard [45], Khan and Glenton [46], Vrielinck *et al*. [47], Lee *et al*. [48], Fogazzi *et al*. [50], Ord [51], Daudon *et al.* [53,54], Prien and Frondel [55], Girometta *et al*. [58], Bird [64], Tyson [66], Freitag and Hruska [71], Frochot and Daudon [72], Sienes Bailo *et al*. [73], Torous *et al*. [74], Díaz-Espiñeira *et al.* [76], Elliot and Rabinowitz [90], Thurgood *et al*. [91], Sun *et al*. [92]
dodecahedral	calcium oxalate dihydrate (C_2_H_4_CaO_6_⋅2H_2_O)	described as prismatic bi-pyramidal, tetragonal prisms or styloid crystals; variants include T-shaped crystals (a crystal with a lateral outgrowth) and interpenetrating twins; dodecahedral crystals 5–53 µm in size	Neuendorf [16], Saffo and Lowenstam [40], Vrielinck *et al*. [47], Lee *et al*. [48], Daudon *et al*. [53,54], Kolo *et al*. [56], Frochot and Daudon [72], Sienes Bailo *et al*. [73], Díaz-Espiñeira *et al.* [76], Elliot and Rabinowitz [90], Sun *et al*. [92], Hackett and Khan [93], Hartl *et al*. [94]

The chemical nature of the six morphotypes of microcrystals in *C. frondosa* was inferred from morphology [16,48] and a combination of solubility, EDX analysis and Raman spectroscopy and classified into three main types: uric acid (cuboidal, barrel-shaped, rhombic, rhombohedral and rosette-shaped microcrystals), calcium carbonate or calcite (cylindrical microcrystals and related variants) and calcium oxalate dehydrate or weddellite (octahedral and dodecahedral microcrystals) (table 3 and figure 6). Based on solubility in an alkaline solution and morphology (including the interior angle where applicable), cuboidal and rhombic microcrystals were classified as uric acid following Lee *et al*. [48]. Moreover, Raman peaks of these microcrystals are consistent with uric acid [97]. Barrel-shaped, rhombohedral and rosette-shaped microcrystals were also determined to be uric acid based on their morphology. In support of this, microcrystals similar to those observed in *C. frondosa* (figure 6) are well documented as uric acid crystals in mammalian urine samples and urinary calculi [15,16,64,67,68,70,75]. However, cuboidal and rhomb-shaped microcrystals (i.e. rhombic and rhombohedral) in *C. frondosa* were challenging to identify because these morphologies can be associated with several types of chemical compounds. For example, rhomb-shaped morphology (typically rhombohedral) can be found in both calcium oxalate monohydrate (whewellite) and calcium carbonate crystals as described by Hartl *et al*. [94], Rademaker and Launspach [98] and Yuan *et al*. [99]. Rhombic or rhombohedral crystals observed here had an interior angle of 62 ± 6°, which was closer to the angle reported for uric acid (i.e. 65°) [55], than for calcium oxalate monohydrate (i.e. approx. 70°) [94] and calcium carbonate crystals (i.e. approx. 80°) [98,99]. The morphology (shape and interior angle), coupled with solubility tests, suggests that rhomb-shaped crystals were also uric acid (table 3 and figure 6). Furthermore, instances of multi-layered rhombic crystals documented in the present study correspond to uric acid reported by Kleeberg *et al*. [70] and Freitag and Hruska [71].

The use of EDX analysis showed that a major chemical component of cylindrical microcrystals consisted of calcium and, combined with their morphology, support their classification as calcium carbonate following Seifan *et al*. [57] and Qin *et al*. [59]. Characteristic Raman peaks (e.g. 1085 cm^–1^) further reinforce this chemical identity [100,101] with trace amounts of another chemical compound (e.g. 1649 cm^–1^) (figure 2C). Having faceted ends and a rough surface, those microcrystals in *C. frondosa* also resemble pineal deposits and ear stones (otoconia) in mammals [52,79,82,83]. Otoconia exhibit a dumbbell-shaped internal structure [83], which morphologically resembles the internal structure of cylindrical microcrystals in *C. frondosa*. Cross-shaped microcrystals in *C. frondosa* were also considered to be calcium carbonate based on those described by Pan *et al*. [87] and Laipnik *et al*. [88] who used EDX and Fourier transform infrared spectroscopy (FTIR) analyses. Similar to the present findings in *C. frondosa*, otoconia in rats and humans also exhibited misaligned and deformed calcite crystals and otoconial intergrowth forming a cross-shaped structure, akin to the phenomenon of twinning in crystals [77,78]. Octahedral and dodecahedral crystals in *C. frondosa* showed morphological features (see table 3) supporting their classification as calcium oxalate dihydrate [48], especially when combined with EDX analysis and Raman spectroscopy. EDX spectra indicated that they were chiefly composed of calcium, carbon and oxygen, matching those by Kolo *et al*. [56] and Girometta *et al*. [58], while Raman spectra exhibited peaks (e.g. 1468 cm^–1^) that are characteristic of calcium oxalate dihydrate [102,103] with trace amounts of another chemical compound (e.g. 1649 cm^–1^) (figure 2D–E).

Overall, microcrystals in *C. frondosa* share similarities with those found in other animal taxa. For instance, all crystalline morphologies of uric acid and calcium oxalate dihydrate in *C. frondosa* resemble urine and urinary calculi of humans, referred to as crystalluria or urinary crystals (figure 6) [14–16,48,53,54,67–71,75]. Cylindrical calcium carbonate (as laminar calcite) and dodecahedral calcium oxalate dihydrate microcrystals were found in the urinary system of horses [44,76]. A variation of the cylindrical calcium carbonate microcrystal with a shorter body was observed in *C. frondosa*, which was morphologically similar to crystals reported in the uterine fluid of chickens [81]. Besides the urinary system of humans, octahedral calcium oxalate dihydrate microcrystals were also detected in rodents [43,46], dogs [45] and pigs [47] and horses [76]. Dodecahedral calcium oxalate dihydrate crystals were reported from the thyroid gland of humans [93]. Among invertebrates, this microcrystal occurred in the renal sac of tunicates [40] and rhombic uric acid crystals were identified in the subcuticular nodule of lobsters [42].

While they have never been formally documented as such, microcrystals have likely been detected before in holothuroids and other echinoderms. For example, ‘crystal cells’ have been described as a type of coelomocyte found in 41% of sea cucumber species (see review by Queiroz *et al*. [104]) and in several species of sea stars and sea urchins [105–107]. Their shape and size (15–22 µm length in *C. frondosa*) [108,109] correspond to the rhomb-shaped uric acid microcrystals outlined here. Besides being individually encapsulated by phagocytes, the fact that these crystals can be free (i.e. not encapsulated) or encapsulated in groups suggests that their origin and function was previously misattributed as crystal cells. Future investigations should revisit the nature and morphological diversity of ‘crystal cells’ described as coelomocytes in echinoderms.

In mammals, the focus of microcrystal studies has been on urine and urinary calculi [76], whereas in invertebrate taxa, the observations of microcrystals appear to have been largely opportunistic in nature [40–42]. The corporeal distribution of microcrystals provided here for *C. frondosa* paves the way for future more in-depth studies in echinoderms.

**Figure 7.**
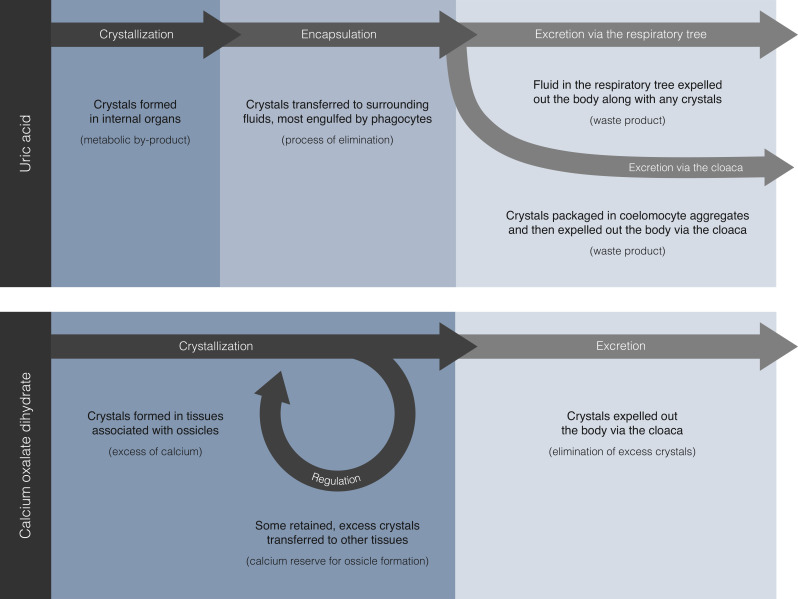
Suggested pathways of crystallization and excretion of uric acid and calcium oxalate dihydrate microcrystals in *C. frondosa*. A pathway for calcium carbonate microcrystals was not apparent due to their limited distribution within the body.

Since the uric acid microcrystals in tissues of *C. frondosa* were more often not encapsulated by coelomocytes and, inversely, those encapsulated were usually found in bodily fluids, the site of synthesis is suspected to be in the former (figure 7). Once formed, crystals of uric acid can remain insoluble in slightly alkaline solutions, which explains their occurrence in bodily fluids with a pH of approximately 7.6 in *C. frondosa* (electronic supplementary material, table S1). In echinoderms, soluble (i.e. non-crystalline) forms of uric acid and other compounds such as ammonia, amino acids and urea were previously detected [110–112], which supports the interpretation that uric acid in its crystalline form is likely a by-product of metabolism in this phylum. Within the body of the sea cucumber, microcrystals were likely transferred from tissues to bodily fluids to be packaged inside coelomocytes and, eventually, excreted out of the body. A higher prevalence of these microcrystals in the perivisceral coelomic fluid and the fluid of the respiratory tree suggests that uric acid tends to accumulate there. This agrees with the presence of soluble forms of uric acid in the coelomic fluids and surrounding tissues of sea urchins, sea stars and sea cucumbers as previously reported by Wilber [110] and Lewis [111]. The packaging of more than one microcrystal in a phagocyte and sometimes associated with other foreign particles supports the notion that encapsulation is part of the process of elimination. Foreign particles were shown in *C. frondosa* and other echinoderms to be trapped by the coelomocytes in either the hydrovascular or perivisceral fluids and, eventually, expelled out of the body [108,109,113]. The same pattern of expulsion seems to be involved with undesirable microcrystals in the body in *C. frondosa*. However, not only were microcrystals expulsed by large aggregates of coelomocytes through the cloaca with other waste material, but individually inside phagocytes through the respiratory tree (figure 7). Furthermore, these microcrystals (e.g. crystals of uric acid) seem to be able to pass through the thin epithelium into the lumen of the respiratory tree and, ultimately, exit from the respiratory tree through the process of intaking and discharging water that is required for respiration (figure 7). Encapsulation likely facilitates the removal of these uric acid microcrystals individually or in groups, in the same way that foreign particles or pathogens are removed with coelomocyte aggregates [108,114].

Regarding calcium oxalate dihydrate, the pathway of its crystallization, usage and excretion may be linked to the regulation of calcium within the body of *C. frondosa* (figure 7). In living organisms, oxalic acid and its salts (e.g. calcium oxalate and sodium oxalate) are end products of metabolism [13,115]. Serving as a potential reservoir of calcium in calcium-rich tissues, the crystallization of calcium oxalate might be related to the maintenance of ionic equilibrium [7,13]. In particular, we speculate that these crystals may be retained at moderate levels in these ossicle-associated tissues (e.g. the integument, tentacles) as a source of calcium for the formation of new ossicles, especially if these tissues are damaged and under repair (figure 7). In holothuroids, calcium oxalate dihydrate crystals may be involved in ionic regulation by serving as a calcium reserve for the construction of ossicles (figure 7), which is a dynamic biochemical process involving the transformation of amorphous calcium carbonate (ACC) into anhydrous ACC, and then into calcite, with the incorporation of magnesium and some organic inclusions into the three-dimensional calcite stereom [32,33,35,116]. Any excess of these crystals is likely transferred to other internal body parts (e.g. muscles). However, the elevated prevalence of these crystals in the cloaca of *C. frondosa* suggests that, when in excess, they may also be eliminated from the body (figure 7).

With respect to cylindrical calcium carbonate crystals (and related variants), a pathway was not fully apparent in *C. frondosa* given that they were only detected in the tissue of the respiratory tree. Given that respiration in holothuroids involves the intake of seawater, calcium carbonate crystals may be formed by microbes [84,86,87] and, subsequently, lodged in the tissue of the respiratory tree of *C. frondosa*. As the case may be for uric acid crystals, any crystals of calcium carbonate in the fluid of the respiratory are likely to exit the body through the process of respiration (i.e. the intake and discharge of water).

Overall, the similarities among the diversity of microcrystals found here in a holothuroid echinoderm and those reported in chordates (mostly mammals) suggest that their synthesis, appearances and pathways of elimination were conserved in the animal kingdom. This study further demonstrated that the occurrence of microcrystals was geographically widespread across populations of *C. frondosa* and taxonomically ubiquitous in holothuroids as shown in four different species. If elevated concentrations of microcrystals in the body of a holothuroid (or other taxon) can be linked to stressor(s), then microcrystal levels (or types) could be added to the repertoire of useful stress biomarkers, e.g. alongside coelomocytes and cortisol [113,117].

## Data Availability

Data supporting this study can be found in the supplemental material [118].
